# Assessment of self-reported prevalence, vaccination status, knowledge and behavioral determinants of hepatitis B and D in Pakistan: a cross-sectional study

**DOI:** 10.3389/fmicb.2026.1748793

**Published:** 2026-02-12

**Authors:** Sajid Ur Rahman, Yanfei Gao, Fazal Noor, Munib Ullah, Hanif Ur Rahman, Abdul Qadeer, Tariq Ullah, Xiumei Chi, Junqi Niu, Saeed Hamid, Zhenfeng Zhang

**Affiliations:** 1 School of Public Health and Emergency Management, School of Medicine, Southern University of Science and Technology, Shenzhen, China; 2 Livestock and Dairy Development Department (Research Wing), Peshawar, Pakistan; 3 Department of Clinical Studies, Faculty of Veterinary and Animal Science, PMAS-Arid Agriculture University, Rawalpindi, Pakistan; 4 Virology Section, CMB Veterinary Research Institute, Peshawar, Pakistan; 5 Department of Cell Biology, School of Life Science, Central South University, Changsha, China; 6 Medical D Unit, Department of Medicine, Ayub Teaching Hospital, Abbottabad, Pakistan; 7 Department of Hepatology, Center of Infectious Diseases and Pathogen Biology, The First Hospital of Jilin University, Changchun, China; 8 Department of Medicine, Aga Khan University, Karachi, Pakistan

**Keywords:** control, HBV/HDV, knowledge, Pakistan, self-reported prevalence

## Abstract

In Pakistan, hepatitis B virus (HBV) and hepatitis D virus (HDV) infections remain a major concern. This study assessed the self-reported prevalence, vaccination status, knowledge, risk factors, attitude, and behavioral practices related to both viruses in Pakistan, along with the impact of stigma and cultural norms on disease perception and management. A cross-sectional study was conducted using an online survey, and the data was collected from 980 general Pakistani public (mostly 18–34 age) from January 15 to March 15, 2025. Among respondents, 79.2% were male and 20.8% female, while 58.5% did not know hepatitis (*p >* 0.05). Only 7.3% reported HBV/HDV self-reported diagnosis, lower than previous studies (16.6%), but 21.3% had a family history, indicating higher self-reported prevalence (*p <* 0.05). Key risk factors included low vaccination coverage (26.4%, χ^2^ = 41.17, *p <* 0.001), chronic infection (χ^2^ = 16.57, *p <* 0.001), needle-sharing (9.2%), unlicensed dental (10.6%) and medical (19.3%) procedures, and poor glove usage (14.8%) (*p <* 0.05). Limited public awareness (35.1%) was noted as the major barrier, while 38.4% of respondents acknowledged the presence of stigma. Participants lack adequate knowledge, gaps in vaccination, risky practices, and stigma persist. A comprehensive public health approach is essential to curb HBV/HDV in Pakistan.

## 1 Introduction

Recent studies have estimated that around 5–15% of individuals with chronic hepatitis B virus (HBV) infection are also coinfected with hepatitis D virus (HDV) (Stockdale et al., 2020). HBV and HDV co-infection causes the most severe form of viral hepatitis, chronic hepatitis D (CHD), which often leads to accelerated progression to cirrhosis, hepatic decompensation, and hepatocellular carcinoma compared to chronic HBV mono-infection (Hughes et al., 2011). Although our understanding of the global HDV prevalence remains incomplete, it is apparent that it is unevenly scattered, with significant hotspots in regions such as Mongolia, Pakistan, Central Asia, Middle East, and Eastern Europe (Khodjaeva et al., 2019). Considering that the hotspots are mostly in developing countries and HDV tests are rarely implemented, the actual global burden of HDV may be significantly underestimated (2024). It is estimated that at least 12 million people globally are living with HDV, though the actual number may be higher due to inadequate testing, particularly in regions with higher prevalence (Da et al., 2021). This suboptimal testing results in the diagnosis of only 20–50% of individuals infected with HDV (Negro and Lok, 2023). According to a previous study, Mongolia shows the highest HDV prevalence rate of 61%, while Pakistan with 16.6% is in second place. Israel, Greece, Saudi Arabia, and Italy showed 5.4, 4.7, 4, and 3.4% prevalence, respectively (Razavi-Shearer et al., 2024).

Estimating the population-level prevalence of viral hepatitis especially HDV is challenging due to substantial biases characteristic in many studies. In most of the countries, national serosurveys are limited, and mostly studies are conducted within particular risk groups or among diagnosed individuals seeking care or long-term treatment (Lazarus et al., 2023). As a result, these studies tend to misjudge the exact prevalence compared to the overall population. This problem is further impaired by the fact that numerous regional serosurveys are conducted in high HDV prevalence areas, which skews the prevalence of national estimations (Stockdale et al., 2020). Additionally, the lack of standardized tests for anti-HDV and the absence of HDV RNA data in many HBV studies further complicate accurate prevalence estimation. Moreover, at the national level, reflex testing-automatically testing all HBsAg+ specimens for anti-HDV at the laboratory level is not regularly implemented. Despite having robust patient registry systems, the lack of reflex testing leads to missed diagnoses of HDV. For example, recent data from different centers in Spain indicate that implementing reflex testing regularly led to a 5-fold increase in the number of HDV-infected individuals (Palom et al., 2022).

Studies based on epidemiological data suggests a decrease in the prevalence of both HBV and HDV, especially in developed countries, with HBV control through vaccination being an important factor. However, the situation remains challenging in developing countries like Pakistan, where inadequate funding and lack of preventative measures impede the control of HBV (Butt et al., 2014). In Pakistan, especially in rural areas, HBV prevalence rates are alarmingly high, ranging from 16 to 57%, and positive individuals of HDV/HBV co-infection are notably more common among young males, particularly in Sindh province (Abbas, 2012). Numerous studies from Pakistan focus on anti-HDV prevalence among HBsAg-positive patients, but these studies do not provide a precise representation of the true viral hepatitis epidemiology (Abbas, 2012; Jalil et al., 2016; Jamil, 2016). Moreover, the vaccination status against HBV is also very low in developing countries, but in Pakistan there is now a reasonable coverage of 50% in infants and around 30% in adults, although this vaccination coverage remains low. On the other hand, limited health knowledge can prevent patients understanding of the importance of regular treatment, while socioeconomic barriers restrict access to required medications and healthcare. This problem is very common in many countries, especially in some Asian countries such as India, Pakistan, Bangladesh, Mongolia, and most of the African countries, where people do not follow the doctor’s protocol properly (Chirambo et al., 2019; Chatha et al., 2020; Ma et al., 2022; Baryakova et al., 2023).

Furthermore, as a protective and preventive measure, spreading knowledge is essential to curb the quick spread of the viral infections especially HBV/HDV. In this regard, both the media, scientific communities, and health-workers should closely collaborate and work together to shape the attitudes of all portions of society. The objective is to control the disease via appropriate practices informed by the knowledge, attitudes, and perceptions surrounding HBV/HDV. A community combined knowledge, attitudes, and perceptions play a significant role in determining how far a virus may spread within a given area (Khattak et al., 2022). Thus, this study was designed to estimate the self-reported HBV/HDV diagnosis, knowledge, risk factors, attitudes, behavioral practices, and perceptions of the Pakistani population regarding HBV/HDV to inform public health policies. By identifying the existing knowledge gaps and misconceptions, this study seeks to offer insights that will help shape targeted educational campaigns, improve community engagement, and strengthen preventive measures against HBV/HDV in Pakistan. Understanding these aspects are important for designing and implementing effective public health interventions, ultimately contributing to the broader goal of safeguarding public health in the face of infectious diseases.

## 2 Methods

### 2.1 Study design

From January 15, 2025 to March 15, 2025, we conducted cross-sectional web-based research with the general Pakistani population. According to the Pakistan Bureau of Statistics 2023, Pakistan’s population is 241.49 million. The study was open to all Pakistanis divided into different categories aged under 18–24, 25–34, and 45 and above, who could understand English. Based on 5% response distribution and 95% margin of error, we calculated the sample size as 385 respondents. However, to achieve more precise and reliable results, we collected 980 responses for the survey.

### 2.2 Study variables

The survey included 48 knowledge-based questions with different response options to assess different indices about HBV/HDV among the Pakistani population. The knowledge score ranged from low to high. Demographic variables included age (categorized as under 18–24, 25–34, and 35 and above years), gender (male, female) or prefer not to say, and place of residence within Pakistan (Khyber Pakhtunkhwa, Islamabad Capital Territory, Punjab, Sindh, Baluchistan, and Gilgit-Baltistan). Additionally, urban or rural residency, employment status, and education level (categorized into primary education, secondary education, and higher education) were recorded. The survey also comprised six questions of medical and family history about HBV/HDV, nine questions about risk factors, five questions of knowledge and awareness of HDV, nine questions evaluating attitude and practices related to disease prevention, four questions related to behavioral practices, and five open-ended questions about HDV screening, government or healthcare measures, biggest barriers of HBV/HDV prevention, awareness, and effect of cultural or societal norms for hepatitis prevention.

### 2.3 Tool for data collection

Data were collected through an online survey. To confirm the reliability of data, the survey accepted only one response per participant, verified via email. Respondents could complete and submit the survey using either a computer or a mobile device. Moreover, to ensure internal consistency, Cronbach’s alpha value in both pilot survey data and final data were calculated and compared, which exceeds the threshold of 0.69 for all the constructs. Cronbach’s alpha values of 0.82, 0.77, 0.75, 0.76, and 0.73 were obtained for the constructs of family history about HBV/HDV, risk factors, knowledge and awareness of HDV, attitudes and practices, and behavioral practices related to healthcare and prevention, respectively exceeding the standard benchmark of 0.70, indicating strong reliability.

The questionnaire was divided into seven sections including demographic characteristics, such as age, gender, residence, education, occupation, and monthly household income. The second section, which comprises medical and family history and has six multiple-choice questions designed to understand the family history about HBV/HDV. The third section had nine risk factors questions, while the fourth section contained five questions based on knowledge and awareness of HDV. The fifth section was contained attitudes and practices about HBV and HDV, while the sixth section was based on behavioral practices related to healthcare and prevention. In last seventh section, we asked 6 open-ended questions about HBV/HDV. Different terms were given to respondents for each question. The survey was written in English.

### 2.4 Procedure for data collection

In the current survey, we did not collect or required the personal information, such as names, addresses, or other identifiable details. All the participants were invited to complete a structured questionnaire via a dedicated Google Forms link (see Supplementary material S1). There was no exact recruitment process for the participants, and the survey was distributed through different platforms, including WeChat, WhatsApp, and Facebook. The link was shared in numerous WeChat, WhatsApp and Facebook groups, with group managers and members encouraged to further distribute it to maximize participation. Before proceeding with the survey, each participant was required to provide informed consent by agreeing to the following statement: “I have read and understood the objectives of this study and willingly agree to participate by providing my responses in a rational manner.” Respondents then completed the questionnaire and submitted their responses through the platform. This was a general survey; therefore, no incentives were offered to the participants. To ensure the reliability of data of the current survey, all questions were mandatory.

### 2.5 Data analysis

Replies less than 75% of the core questions were deemed incomplete and excluded from later analysis. The responses collected via Google Forms were exported to Microsoft Excel. Statistical analysis was conducted in Statistical Package for the Social Sciences (SPSS) v27.0 statistical software (SPSS Inc., Chicago, IL, United States) and Graph Pad Prism, v9.0 (Grappa Software, San Diego, CA, United States). The variables were coded in SPSS, and missing data were examined using Missing Value Analysis to ensure data completeness. Factors linked with HBV/HDV vaccination status, self-reported prevalence, knowledge, attitude, and perception were discovered using chi-square and linear-by-linear association analysis. Binomial logistic regression analysis was used to examine the association between participants demographic characteristics and associated risk factors. The performance of model was evaluated by using omnibus chi-square test, Nagelkerke R^2^, Hosmer–Lemeshow goodness-of-fit test, and classification accuracy. Odds ratios with 95% confidence intervals were reported. Descriptive statistics such as frequency and percentage were employed to demonstrate the demographic features of the research participants. A value with *p <* 0.05 was set to determine statistical significance.

## 3 Results

### 3.1 General characteristics of the respondents

In the final analysis, 980 participants were recruited, with 79.2% males, while just 20.8% were females. About 43.9% of the respondents were from Punjab province, while 20.2% from Sindh, 14.4% from Khyber Pakhtunkhwa, 10.6% from Baluchistan, 5.7% from Islamabad (the capital territory), and 5.2% of citizens were belonged to Gilgit-Baltistan. Most respondents were 18–24 (70.9%) years old. The remaining 20.9% were 25–34 years, while only 8.2% participants were 35+ years old. Table 1 provides an overview of demographic characteristics of the research respondents.

**Table 1:** Demographic descriptions of participants (*n* = 980).

S. No	Variable	Unique values	Frequency	Percentage (%)
1	Gender	Male	776	79.2
		Female	204	20.8
2	Age	18–24	695	70.9
		25–34	205	20.9
		35 years and above	80	8.2
3	Province of residence	Punjab	430	43.9
		Sindh	198	20.2
		Khyber Pakhtunkhwa	141	14.4
		Baluchistan	104	10.6
		Islamabad Capital Territory	56	5.7
		Gilgit-Baltistan	51	5.2
4	Education level	Primary education	19	1.9
		Secondary education	36	3.7
		Higher education	925	94.4
5	Residence type	Urban	541	55.2
		Rural	439	44.8
6	Occupation	Healthcare worker	223	22.8
		Office worker	51	5.2
		Students	706	72.0
7	Monthly household income (PKR)	20,000–50,000	97	9.9
		50,001–100,000	326	33.3
		100,000–200,000	274	28.0
		>200,000	283	28.9

### 3.2 History of respondents, vaccination status and self-reported prevalence of HBV/HDV

In this section, first the participants were asked about their diagnosis of HBV/HDV. The results in Table 2 shows that 92.7% (908) of respondents replied “No,” while 7.3% (72 peoples) out of 980 said “Yes.” According to this survey about 72 positive cases with a self-reported prevalence rate of 7.34% were observed in all the respondents from different areas of Pakistan. The family history showed 209 positive cases with a self-reported prevalence rate of 21.32% out of 980 participants as shown in Table 2. The main reasons for the high self-reported infections maybe smaller number of vaccinations against HBV (56.5%, 118/209) (χ^2^ = 41.17, *p <* 0.001), chronic illnesses (χ^2^ = 16.57, *p <* 0.001), less focuses on the symptoms related to hepatitis, and the currently available treatments (Supplementary Table S1). During analysis, we observed a significantly higher self-reported prevalence in individuals having symptoms such as fatigue, abdominal pain, swelling, loss of appetite, nausea, or vomiting among individual from HBV positive families (52.6%, 110/209) compared to those from HBV negative families (38.4%, 296/771), with a statistically significant association (*p <* 0.05). The complete details of the medical and family history, along with the reasons for the higher self-reported prevalence of HBV/HDV, are summarized in Table 2.

**Table 2:** Overall self-reported prevalence related to respondents medical, and family history for HBV/HDV.

S. No	Questions/variable	Options	Frequency	Percentage (%)
1	Have you been diagnosed with hepatitis B, or hepatitis D?	Yes	72	7.3
		No	908	92.7
2	Has anyone in your family been diagnosed with hepatitis B, or hepatitis D?	Yes	209	21.3
		No	771	78.7
3	Have you been vaccinated against hepatitis B?	Yes	259	26.4
		No	721	73.6
4	Do you have any chronic illnesses (e.g., diabetes, hypertension)?	Yes	78	8.0
		No	902	92.0
5	Are you currently undergoing treatment for hepatitis?	Yes	14	1.4
		No	966	98.6
6	Have you experienced any of the following symptoms in the last 6 months (Fatigue, Jaundice, yellowing of skin/eyes, abdominal pain/swelling, nausea, or vomiting?)	Yes	406	41.4
		No	574	58.6

### 3.3 Knowledge of the participants about HBV/HDV

**Figure 1:**
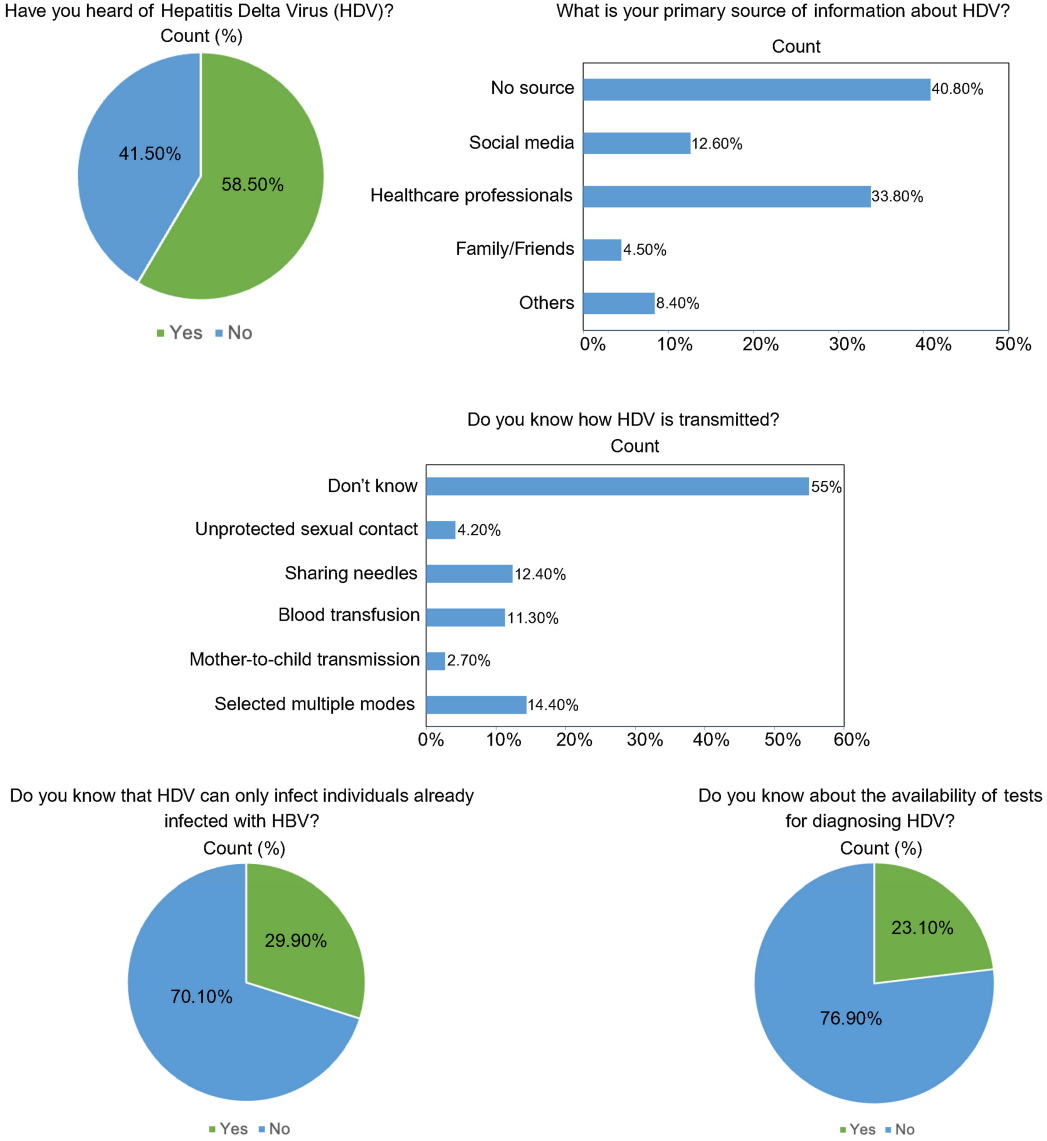
Knowledge and awareness of the participants about HDV, its source of knowledge, transmission route, infectivity, and diagnostic tests.

Figure 1 illustrates participants’ responses to questions assessing their knowledge and awareness of HBV/HDV. Out of 980 male and female participants, 58.5% (573) lack sufficient knowledge about hepatitis viruses. Several questions received incorrect answers, such as 40.80% (400) of participants stated there is no primary source of information they know about HBV/HDV. Furthermore, about 55% of participants have no understanding of HDV transmission. Only 29.90% (293) were aware that HDV can only infect individuals already infected with HBV. As shown in the last panel of Figure 1, 76.90% (754) of participants lack knowledge of the diagnostic tests for HDV.

### 3.4 Risk factors and behavioral practices related with self-reported HBV/HDV

The data in Table 3 show that several reported risk factors and behavioral practices were related with self-reported HBV/HDV in the current survey. Out of 980 participants, 19.3% (189) had received medical procedures from unlicensed healthcare providers. Furthermore, about 33.2% (325) of respondents have a history of traveling outside the country or province for work or leisure (Table 3). In total, 4.9% (48) of peoples had a history of shared or reused shaving blades at barbershops, while 26.6% of participants had a history of living and working together with the people who already had HBV or HDV. Moreover, 9.2% (90) of participants have claimed that they used shared needles, syringes and other medical equipment’s, and 10.6% (104) respondents had a history of experienced dental treatment in unlicensed clinics (Table 3). In this case, individuals from HBV/HDV positive families exhibited several risk factors compared to those from non-affected families, including history of blood transfusion (11.0%, 23/209 vs. 5.3%, 41/771), and exposure to unlicensed healthcare providers (26.8%, 56/209 vs. 17.3%, 133/771), with all differences showing statistical significance (*p <* 0.05) (Supplementary Table S1).

**Table 3:** Risk factors, behavioral practices and their association with the self-reported prevalence of HBV/HDV in Pakistani population (*n* = 980).

S. No	Questions/variable	Options	Frequency	Percentage (%)
1	Have you ever received a blood transfusion?	Yes	64	6.5
		No	916	93.5
2	Have you ever undergone any surgical procedures?	Yes	261	26.6
		No	719	73.4
3	Have you ever shared needles, syringes, or other medical equipment?	Yes	90	9.2
		No	890	90.8
4	Have you ever undergone dental treatment in an unlicensed clinic?	Yes	104	10.6
		No	876	89.4
5	Do you frequently visit barbershops or salons for shaving or grooming?	Yes	605	61.7
		No	375	38.3
6	Have you received treatment or medical procedures from unlicensed healthcare providers?	Yes	189	19.3
		No	791	80.7
7	Do you frequently travel outside your province or country for work or leisure?	Yes	325	33.2
		No	655	66.8
8	Have you ever shared or reused shaving blades at barbershops?	Yes	48	4.9
		No	932	95.1
9	Have you lived or worked with someone diagnosed with hepatitis B, or D?	Yes	261	26.6
		No	719	73.4
10	Do you insist on using new syringes and needles during injections or medical procedures?	Yes	895	91.3
		No	85	8.7
11	Do you avoid sharing personal items such as razors or toothbrushes?	Yes	900	91.8
		No	80	8.2
12	Do you verify the cleanliness of medical and dental equipment before treatment?	Always	691	70.5
		Never	64	6.5
		Sometimes	225	23.0
13	Do you wear gloves while handling blood or other body fluids at work (if applicable)?	Yes	835	85.2
		No	145	14.8

We further asked about the behavioral practices related to healthcare and prevention. In total, 8.7% (85) of respondents replied that they never insist on using new syringes and needles during injection or medical procedures. Moreover, 8.2% (80) respondents answer that they are not avoid sharing personal items such as razors or toothbrushes, and 6.5% (64) replied that they never verify the cleanliness of medical or dental equipment before treatment, while 23.0% (225) replied they sometime verify it. When handling blood or other body fluids during work, 14.8% (145) people responded that they do not wear gloves or washed their hands properly with disinfectants (Table 3). However, some risk factors were statistically non-significant such as surgical procedures (*p* = 0.126), needle sharing (*p* = 0.194), dental treatment in unlicensed clinics (*p* = 0.645), frequent barbershop visits (*p* = 0.518) and reused shaving blades (*p* = 0.419).

### 3.5 Government policies, healthcare strategies, and challenges in the control of HBV/HDV in Pakistan

**Figure 2:**
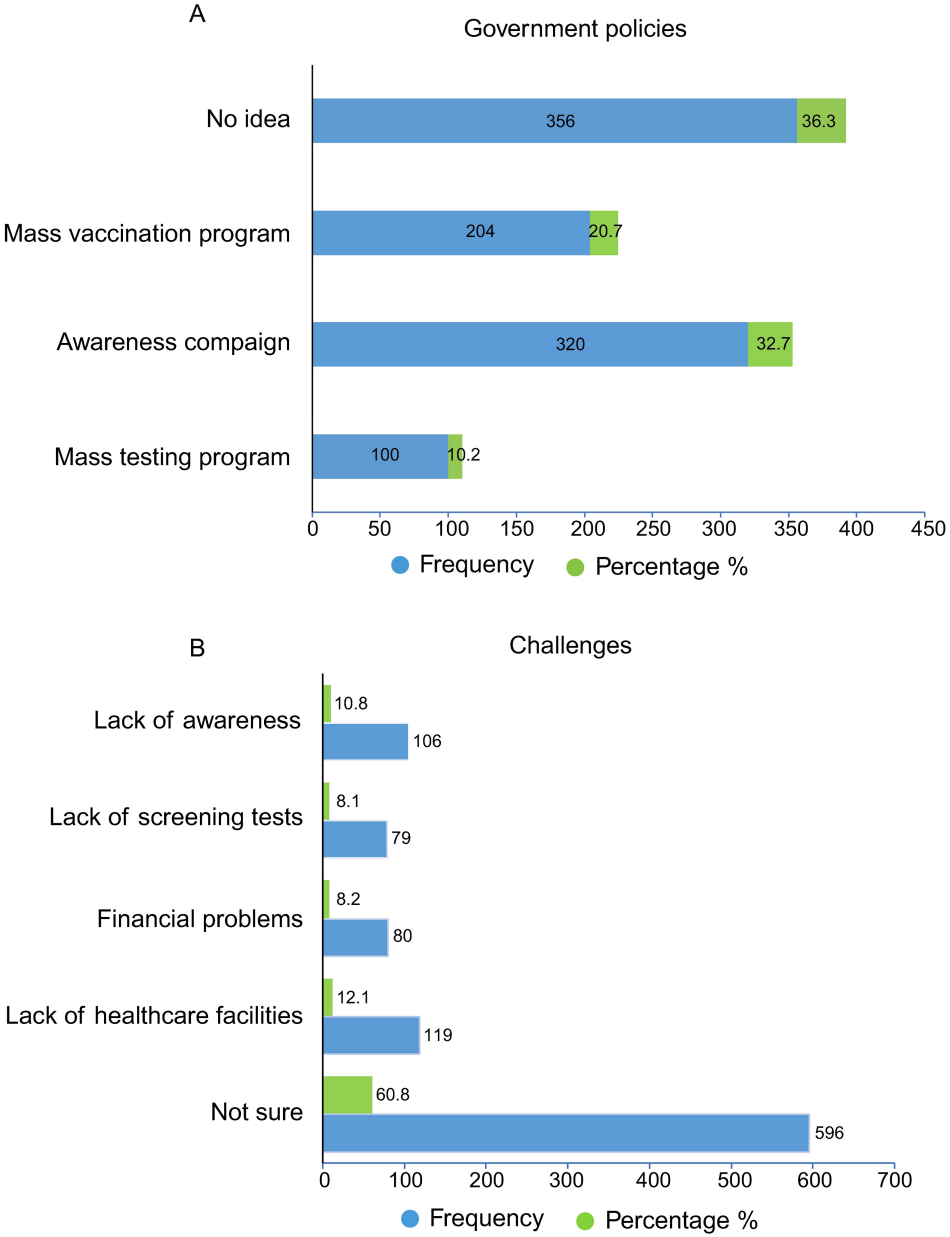
Distribution of respondents’ perspectives on **(A)** government policies, healthcare strategies, and **(B)** key challenges in the control of HBV/HDV in Pakistan.

In Figure 2A, 36.3% (356/980) of respondents reported having no clear idea about what actions the government or healthcare providers should take to control HBV/HDV in Pakistan. In contrast, 10.2% (100/980) suggested that implementing mass testing programs could help control HBV/HDV, while 32.7% (320/980) believed that awareness campaigns maybe effective. Additionally, 20.8% (204/980) of respondents indicated that nationwide mass vaccination programs would be the most effective strategy for HBV/HDV control (Figure 2A).

In the current study, a high percentage of 60.8% (596 out of 980) respondents were not sure about the challenges in accessing hepatitis screening or treatment services (Figure 2B). Moreover, 12.1% (119) have facing lack of healthcare facilities challenge, and 8.2% (80) of respondents think that financial problem is the main challenge in the control of HBV/HDV in Pakistan (Figure 2B).

### 3.6 Perceived cultural and societal barriers to hepatitis awareness in Pakistan

**Figure 3:**
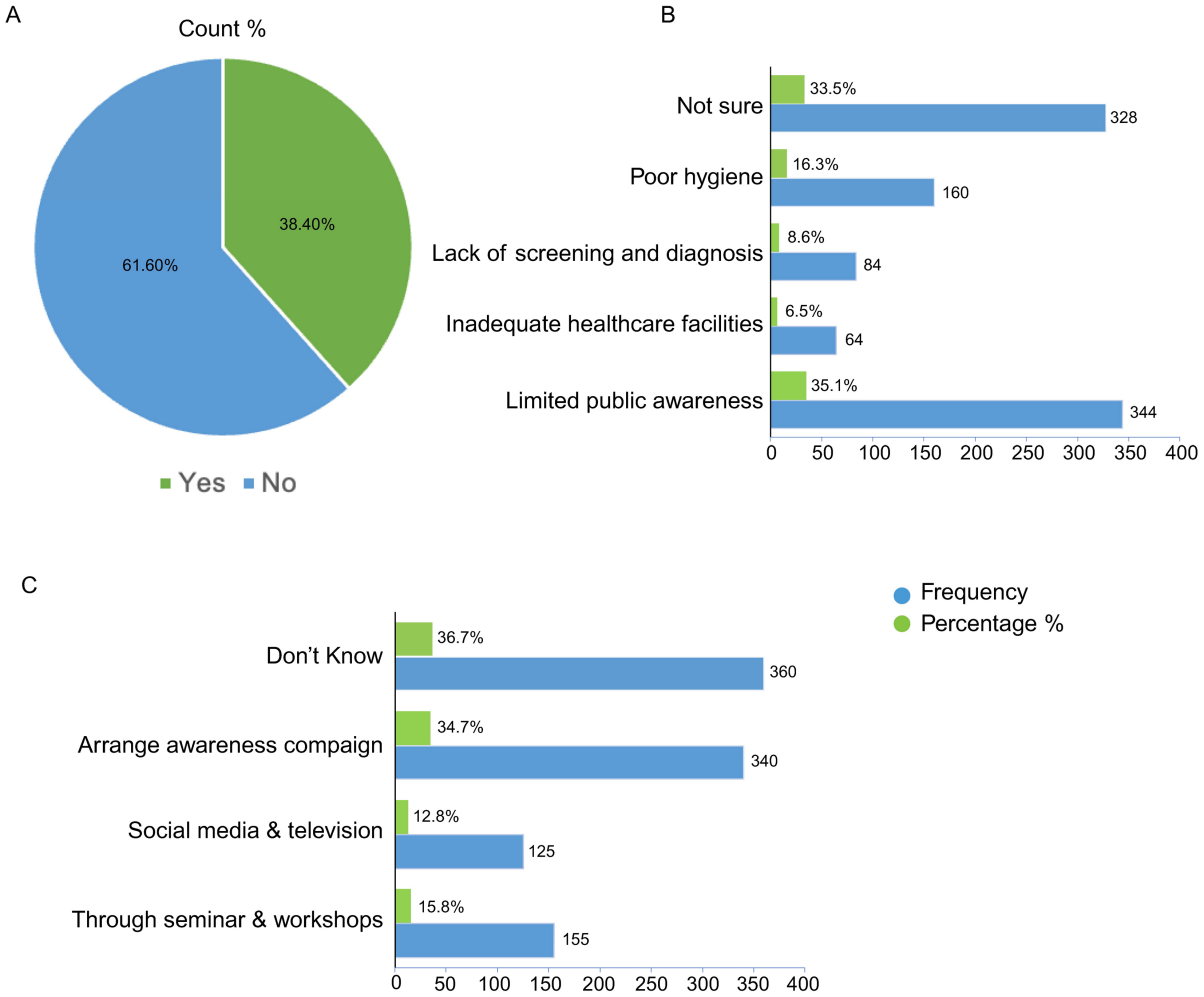
Respondents’ perspectives on the influence of cultural and societal norms on hepatitis awareness and control in the Pakistani community. **(A)** Perceptions of how cultural or societal norms influence the understanding and treatment of hepatitis in the Pakistani community. **(B)** Respondents’ views on the major barriers hindering effective control of HBV/HDV **(C)** Suggested strategies by respondents to mitigate the impact of cultural or societal norms and enhance public awareness and education about hepatitis in Pakistan.

In Figure 3A, our data shows 38.4% (376) respondents thinking that cultural or societal norms affect how hepatitis is perceived or treated in the Pakistani community (Figure 3A). About 35.1% (344 out of 980) respondents claimed that limited public awareness were the main barrier to control HBV/HDV in Pakistan, while 6.5% replied healthcare facilities in the country, 8.6% responded that lack of perfect screening and diagnosis, and 16.3% people replied that poor hygiene is the main barrier to control HBV/HDV in Pakistan (Figure 3B). Remaining 33.5% were not sure about the biggest barrier for this problem (Figure 3B).

To improve awareness for cultural and societal norms and against HBV/HBV in the community, 15.8% (155) peoples think that through seminars and workshop arrangement in the country could improve awareness against HBV/HDV, while 12.8% (125) replied that social media and television could be a best choice to raise awareness in the community (Figure 3C). Furthermore, 34.7% (340 out of 980) respondents replied that arranging awareness campaigns could be helpful in reducing the impact of cultural or societal norms and improve hepatitis awareness in the Pakistani community, still a large number of 36.7% (360) respondents have no idea about this problem (Figure 3C).

### 3.7 Association of knowledge, risk factors, attitude, behavioral practice and perception of Pakistani population toward HBV/HDV

The results presented in Table 4 show that respondents with a family history of HBV/HDV had 89.3% lower odds of reporting an HBV/HDV diagnosis compared to those having no family history. Participants who perceived themselves to be at low risk had 85% lower odds of reporting diagnosis compared to those who perceived high risk. Additionally, participants with low knowledge had 1.94 times higher odds of reporting HBV/HDV diagnosis compared to the respondents having high HBV/HDV knowledge. Participants with a negative attitude shows 43% lower odds of reporting an HBV/HDV diagnosis compared to the positive attitude participants. Similarly, respondents with uncertain awareness had 45% lower odds of reporting diagnosis compared to those who were proactively aware. No statistically significant association was observed between preventive practices and reported HBV/HDV diagnosis (Table 4).

**Table 4:** Binomial regression reporting adjusted odds ratio with 95% CI associated with self-reported HBV/HDV infection.

Variables	OR (95% CI)	*p*-value
Family history of HBV/HDV		
Yes	0.11 (0.07–0.18)	<0.001***
Perceived risk		
Low	0.15 (0.09–0.25)	<0.001***
Knowledge		
Low	1.94 (1.10–3.41)	0.022*
Attitude		
Negative	0.57 (0.33–0.96)	0.034*
Preventive practices		
Low	1.13 (0.68–1.88)	0.636
Awareness		
Uncertain	0.55 (0.31–0.97)	0.040*

Reference categories were: No, High Risk, High Knowledge, Positive attitude, High and Proactive.

*p < 0.05, **p < 0.01, ***p < 0.001.

## 4 Discussion

HBV along with HDV, poses a serious public health challenge, accelerating liver cirrhosis and significantly increasing morbidity and mortality (Alfaiate et al., 2020). In Pakistan, HBV and HDV infections remains a major concern. Alarmingly, awareness regarding HBV/HDV transmission, prevention, and associated risk factors are declining in Pakistan, even among affected individuals. A recent meta-analysis reported Mongolia as having the highest HDV prevalence (61%), while Pakistan also exhibits a substantial burden, with 16.6% of HDV-infected individuals (Razavi-Shearer et al., 2024). Despite this, HBV/HDV testing remains limited in Pakistan, leading to a likely underestimation of the true disease burden. A previous study discussed that a key contributing factor maybe the widespread misconception that HBV/HDV is rare and of minimal clinical significance (Abbas and Abbas, 2024). The current large-scale cross-sectional study aims to enhance the current understanding of HBV/HDV infections in Pakistan and underscore their clinical relevance. By investigating positive individual rates, vaccination status, knowledge levels, risk factors, attitudes, behavioral practices, and public perceptions, this study will serve as a critical foundation for future research and public health initiatives targeting HBV/HDV in Pakistan.

The current data were collected from over 980 individuals, including healthcare workers, students, office employees, and others, with the majority of responses coming from postgraduates. We assessed their perceptions of HBV/HDV, vaccination status against HBV, and the self-reported positive cases of HBV and HDV. Additionally, we examined key risk factors, public attitudes, particularly the stigma associated with HBV/HDV infection and overall knowledge about hepatitis. This study is particularly important because, similar to some other Asian countries, many people in Pakistan do not strictly adhere to the medical guidelines (Chatha et al., 2020; Baryakova et al., 2023). This non-compliance increases the risk of many infections, particularly HBV and HDV, highlighting the urgent need to improve awareness and initiate preventive measures. In the current study, our binomial regression model was statistically significant, good fit and explained appropriately 90.3% variance. Our model shows statistically significant data based on χ^2^ (6) = 934.649, *p <* 0.001 (indicating that the predictors reliably explained our dependent variable). Hosmer-Lemeshow for goodness-of-fit test indicated that the model is a good fit (χ^2^ = 121.628, *p* = 0.310). Moreover, the model has explained 82.0% of the variance (Nagelkerke R^2^ = 0.820), and correctly classified 90.3% of cases.

The respondent was mostly male of about 776 and the remaining 204 out of 980 were females. The participants were belonging to different provinces such as Punjab 430, Khyber Pakhtunkhwa 141, Sindh 198, Baluchistan 104, Gilgit Baltistan 51, and the capital territory Islamabad 56 respondents out of 980. All participants were responded through an online google form. Our findings indicate that some respondents had a moderate understanding of HBV/HDV transmission and prevention, but 754 out of 980 respondents having no or limited HBV/HDV knowledge (Figure 1; Table 4) (Ahmed et al., 2023). According to previous study, the prevalence of HDV among people in Pakistan was 16.6% (Razavi-Shearer et al., 2024). Our study shows a self-reported prevalence of HBV/HDV infection lowered than the previous study. This difference is likely related to differences in study design, sampling strategy, and population characteristics. However, when we asked respondents about their family history and calculated the prevalence, it shows a higher self-reported prevalence rate (Table 2), suggesting possible family clustering and shared risk factors that may not be sufficiently reflected in population-level assessments. The self-reported prevalence of HBV/HDV across demographics are shown in Supplementary Table S2. We find that the higher prevalence was due to a smaller number of vaccinations against HBV (56.5%, 118/209) (χ^2^ = 41.17, *p <* 0.001), chronic illnesses, and less focuses on the symptoms related to hepatitis. Furthermore, the risk factors and behavioral practices such as sharing blades at barbershops, using shared needles and medical/dental equipment’s at unlicensed clinics or pharmacies, and no use of gloves while handling blood or body fluids may also be the causes of higher HBV/HDV prevalence in Pakistan. However, comparisons between previous study and our study are limited by different methods of detection (Razavi-Shearer et al., 2024). Therefore, these findings warrant additional validation by a well-designed serological and longitudinal studies. Overall, given our sample size, we believe our estimate to be closer to true prevalence and propose the burden of HBV/HDV in Pakistan may in fact be much higher than previously believed (Aftab et al., 2018).

Moreover, around 400 participants claimed that they have no source of information available about HBV/HDV. Similar findings have been reported in previous studies conducted in other countries, such as Vietnam, where research at designated clinics and healthcare centers identified significant knowledge gaps among medical students concerning HBV transmission, the associated health risks, and available diagnostic tests (Wang et al., 2016). As illustrated in Figure 1, our study similarly found a positive correlation between respondents’ knowledge and their approach to hepatitis management. A lack of understanding and widespread misconceptions about HBV/HDV transmission, particularly the increased risk of chronic infection when the virus is contracted at birth can lead to missed opportunities for prevention, including vaccination, and may also contribute to the stigma and discrimination faced by individuals living with HBV/HDV. Similar patterns have been observed in studies conducted in Ghana (Osei et al., 2019; Adam and Fusheini, 2020). Additionally, another study highlighted a general lack of information regarding HBV/HDV prophylaxis (Le An et al., 2021). These findings suggest that individuals may not have received adequate education for the prevention of HBV/HDV, which could hinder both the general public and hepatitis patients from effectively managing their health. Enhancing screening among healthy blood donors is crucial for both preventing transmission and assessing the disease burden especially in Pakistan, where approximately 1.5 million people donate blood annually (Zahoor et al., 2021). Despite efforts, challenges such as inadequate healthcare facilities, low socioeconomic status, and limited awareness about HBV/HDV transmission persist (Ali et al., 2011). In response, various institutions in Pakistan have launched initiatives to educate the public about hepatitis especially HBV/HDV. In this case, the National Institute of Health (NIH) in Islamabad has identified key environmental risk factors and implemented training programs for lady health workers and their supervisors, aiming to raise awareness and promote preventive measures to combat the spread of hepatitis. Moreover, this study finds the challenges such as limited public awareness, inadequate screening, low vaccination coverage, and poor healthcare infrastructure hinder effective disease control. The government has implemented strategies such as the Expanded Programme on Immunization (EPI), which includes the HBV vaccine, and the Prime Minister’s Health Program, aimed at improving access to treatment (Hasan et al., 2010). This program targets almost 7.5 million children every year throughout the country and may prevent up to 17% of the childhood mortality. However, about 356 out of 980 (36.3%) peoples lack basic information about the government policies according to our survey. Moreover, some respondents answered that awareness campaigns, mass testing as well as mass vaccination program would be helpful in controlling HBV/HDV.

Cultural and societal norms can significantly impact individual identity and mental health in negative ways (Charlesworth and Hatzenbuehler, 2025; Ring and Lawn, 2025). In many Asian countries, the stigma and cultural perceptions associated with hepatitis remain poorly acknowledged. This study explores how such norms affect the quality of life in individuals infected with hepatitis. As illustrated in Figure 3A, both stigma and prevailing cultural attitudes maybe contribute to a decline in life quality, with respondents reporting high levels of stigmatization among infected individuals. However, the results presented in our study reflect self-reported opinions within the study population and should be interpreted with caution, as they may not represent the larger or whole community. According to the modified labeling theory, internal discrimination associated to a socially devalued condition can have harmful psychological and behavioral effects (Kroska and Harkness, 2008; Noor et al., 2016). Furthermore, self-stigma and cultural or societal pressures have been linked to increased disease symptoms, reduced treatment adherence, increased suicidal ideation, and a substantial decrease in overall quality of life (Vrbova et al., 2018; Armoon et al., 2022). The widespread stigma and sociocultural norms surrounding health conditions pose a significant barrier to effective treatment and rehabilitation, particularly for individuals with HBV or HDV (Eilinghoff et al., 2024). Enhancing the quality of life for people with chronic illnesses requires identifying and implementing effective strategies to raise awareness about the harmful effects of stigma and cultural biases (Barqawi et al., 2025).

Taken together, our study shows that family history, perceived risk, knowledge, attitude, and awareness were significantly associated with self-reported HBV/HDV diagnosis, while preventive practices showed no significant association (Table 4). Our findings propose that differences in awareness, risk perception, and attitudes may influence how individuals recognize, perceive, or report HBV/HDV infection (Wang et al., 2016). Furthermore, our results highlight the potential importance of strengthening education, risk communication, and awareness campaigns to support early detection and engagement with prevention services of hepatitis. However, these associations should be interpreted carefully due to the cross-sectional design and the self-reported nature of the data.

## 5 Study limitations

Several limitations should be considered when interpreting the results of this study. First, the reliance on convenience sampling may leads to selection bias, potentially limiting the representativeness of the samples. Additionally, the online distribution of the survey via platforms such as Facebook, WhatsApp, and WeChat may have excluded individuals with limited internet access, low education level, or lower socioeconomic backgrounds. As a result, the generalizability of the outcomes to the broader population may be limited. Furthermore, since the study focused upon previously published literature available only in English, language bias may have influenced participants understanding, particularly when addressing technical or complex concepts. This could have affected the accuracy and reliability of their responses. Moreover, respondents may have provided socially desirable answers, tailoring their responses to align with perceived expectations rather than expressing their true attitudes and perceptions. Fourth, the study is susceptible to recall bias, as participants self-reported data may have been influenced by flaws or memory distortions.

Additionally, the majority of residents were from urban areas, which may limit the applicability of findings stratified by the factors discussed above. The gender imbalance with 79.2% of participants being male also raises concerns about the generalizability of the results, particularly regarding gender-specific trends, and should be accepted as a key limitation. Moreover, the questionnaire was designed solely in English, which may have excluded less educated people, especially in a developing country context, further compromising the sample’s representativeness.

## 6 Conclusion

Overall, the current study indicate that the Pakistani population have limited knowledge, awareness, and perceptions regarding HBV and HDV. Moreover, gaps in vaccination for HBV, risky practices, and stigma persist. Furthermore, our study may contribute to advancing knowledge about HBV vaccination status among healthcare professionals, the general public, policymakers and others. Besides, it is important to prioritize the quality of life and address the stigmatization of individuals living with chronic illnesses especially viral diseases such as HBV/HDV, with a strong emphasis on their rights, self-respect, and overall physical, mental, and social well-being. A comprehensive public health approach, including mass vaccination, risk management programs, education on cultural norms, and improved hygiene is essential to curb HBV/HDV in Pakistan. The outcomes of this study may highlight the urgent need to improve public education and awareness regarding HBV/HDV and their preventive strategies. Moreover, the findings should be interpreted as indicative insights that may help inform public health department and guide future population-based, or serologically confirmed studies.

## 7 Implications of the study

The present study has important implications for doctors and researchers working in the field of hepatitis. This study provides valuable insights into the epidemiology, public awareness, and systemic challenges surrounding HBV/HDV infections in Pakistan. By identifying the exact prevalence rate of HBV/HDV by population-based studies with serological confirmation, such as intra-family transmission, and assessing immunization coverage and its associated factors, our study highlighted critical gaps for the prevention of the infections. Moreover, the current study sheds light on the public knowledge, attitudes, risk behaviors, and perceptions, which are essential for designing effective awareness and intervention programs for the control of viral infection especially HBV/HDV. Additionally, our study highlighted the role of government and healthcare measures, as well as identifying key barriers such as limited access to screening, vaccination and treatment services, and the universal influence of cultural norms and stigma. The findings of the current study may offer indicative insights for public health policies, highlighting the need for more comprehensive, culturally sensitive, and accessible hepatitis control strategies in Pakistan.

## Data Availability

The original contributions presented in the study are included in the article/Supplementary material, further inquiries can be directed to the corresponding author.
