# Supplementary material for: Assessment of self-reported prevalence, vaccination status, knowledge and behavioral determinants of hepatitis B and D in Pakistan: a cross-sectional study
Source: Front Microbiol. 2026 Feb 12;17:1748793. doi: 10.3389/fmicb.2026.1748793 (PMC12935930; doi:10.3389/fmicb.2026.1748793)
Supplement: Supplementary file 1 [file Table_1.docx]

**Assessment of self-reported prevalence, vaccination status, knowledge and behavioral determinants of hepatitis B and D in Pakistan: A cross-sectional study**

**Informed consent**

Hello everyone, we are conducting a survey titled “Assessment of self-reported prevalence, vaccination status, knowledge and behavioral determinants of hepatitis B and D in Pakistan: A cross-sectional study.” The study results will represent the self-reported prevalence rate, vaccination status, knowledge, attitudes, and perception toward Hepatitis B and D Virus in Pakistan. The survey may take between 10-15 minutes to complete. You will not be asked for any personal information in the survey. You have the right to participate or deny; you can withdraw from replying during your participation. The study will not benefit you financially or in any other way, but policymakers may consider the findings and lead to implementing hepatitis B or hepatitis D viruses’ treatments in Pakistan. Participants under the age of 18 should not take part in the survey. Before proceeding with the survey, you are required to agreeing to the following statement: "I have read and understood the objectives of this study and willingly agree to participate by providing my responses in a rational manner."

#### **Section 1: Demographics**

1. **Age:**
   - ☐ 18–24
   - ☐ 25–34
   - ☐ 35 and above
2. **Gender**:
   - ☐ Male
   - ☐ Female
   - ☐ Prefer not to say
3. **Province of Residence**:
   - ☐ Punjab
   - ☐ Sindh
   - ☐ Khyber Pakhtunkhwa
   - ☐ Baluchistan
   - ☐ Islamabad Capital Territory
   - ☐ Gilgit-Baltistan
4. **Education Level**:
   - ☐ Primary education
   - ☐ Secondary education
   - ☐ Higher education
5. **Occupation**:
   - ☐ Healthcare worker
   - ☐ Office worker
   - ☐ Student
6. **Monthly Household Income** (PKR):
   - ☐ < 20,000
   - ☐ 50001–10,0000
   - ☐ >100,000
   - ☐ 200000-500000
7. **Residence Type**:
   - ☐ Urban
   - ☐ Rural

#### **Section 2: Medical and Family History**

1. **Have you been diagnosed with hepatitis B, or hepatitis D?**
   - ☐ Yes
   - ☐ No
2. **Has anyone in your family been diagnosed with hepatitis B, or hepatitis D?**
   - ☐ Yes
   - ☐ No
   - ☐ Don’t know
3. **Have you been vaccinated against hepatitis B?**
   - ☐ Yes
   - ☐ No
   - ☐ Not sure
4. **Do you have any chronic illnesses (e.g., diabetes, hypertension)?**
   - ☐ Yes
   - ☐ No
5. **Have you experienced any of the following symptoms in the last 6 months?** (Select all that apply)
   - ☐ Fatigue
   - ☐ Jaundice (yellowing of eyes/skin)
   - ☐ Abdominal pain/swelling
   - ☐ Loss of appetite
   - ☐ Nausea or vomiting
   - ☐ None
6. **Are you currently undergoing treatment for hepatitis?**
   - ☐ Yes
   - ☐ No

#### **Section 3: Risk Factors**

1. **Have you ever received a blood transfusion?**
   - ☐ Yes
   - ☐ No
2. **Have you ever undergone any surgical procedures?**
   - ☐ Yes
   - ☐ No
3. **Have you ever shared needles, syringes, or other medical equipment?**
   - ☐ Yes
   - ☐ No
4. **Have you ever undergone dental treatment in an unlicensed clinic?**
   - ☐ Yes
   - ☐ No
5. **Do you frequently visit barbershops or salons for shaving or grooming?**
   - ☐ Yes
   - ☐ No
6. **Have you received treatment or medical procedures from unlicensed healthcare providers?**
   - ☐ Yes
   - ☐ No
7. **Do you frequently travel outside your province or country for work or leisure?**
   - ☐ Yes
   - ☐ No
8. **Have you ever shared or reused shaving blades at barbershops?**
   - ☐ Yes
   - ☐ No
9. **Have you lived or worked with someone diagnosed with hepatitis B, or D?**
   - ☐ Yes
   - ☐ No

#### **Section 4: Knowledge and Awareness of Hepatitis Delta Virus**

1. **Have you heard of Hepatitis Delta Virus (HDV)?**
   - ☐ Yes
   - ☐ No
2. **If yes, what is your primary source of information about HDV?**
   - ☐ No source
   - ☐ social media
   - ☐ Healthcare professionals
   - ☐ Friends/Family
   - ☐ Other
3. **Do you know how HDV is transmitted?** (Select all that apply)
   - ☐ Blood transfusion
   - ☐ Sharing needles
   - ☐ Mother-to-child transmission
   - ☐ Unprotected sexual contact
   - ☐ Don’t know
4. **Do you know that HDV can only infect individuals already infected with Hepatitis B Virus (HBV)?**
   - ☐ Yes
   - ☐ No
5. **Do you know about the availability of tests for diagnosing HDV?**
   - ☐ Yes
   - ☐ No

#### **Section 5: Attitudes and Practices**

1. **Do you believe hepatitis can be prevented through vaccination?**
   - ☐ Yes
   - ☐ No
   - ☐ Not sure
2. **How often do you screen for hepatitis B or other liver conditions?**
   - ☐ Regularly, occasionally (at least once a year)
   - ☐ Never
3. **Are you willing to get tested for HDV if recommended?**
   - ☐ Yes
   - ☐ No
4. **What preventive measures do you take to avoid hepatitis transmission?** (Select all that apply)
   - ☐ Use disposable syringes
   - ☐ Avoid sharing personal items (razors, toothbrushes)
   - ☐ Practice safe sex
   - ☐ Ensure proper sterilization of medical/dental equipment
5. **If diagnosed with HDV, would you share your condition with family and friends?**
   - ☐ Yes
   - ☐ No
6. **Do you think people with hepatitis face social stigma in your community?**
   - ☐ Yes
   - ☐ No
7. **Would you be willing to participate in a hepatitis awareness campaign?**
   - ☐ Yes
   - ☐ No
8. **Do you use boiled or filtered water for drinking at home?**
   - ☐ Yes
   - ☐ No
9. **How often do you visit healthcare facilities for regular check-ups?**
   - ☐ Every 6 months
   - ☐ Once a year
   - ☐ Rarely
   - ☐ Never

#### **Section 6: Behavioural Practices Related to Healthcare and Prevention**

1. **Do you insist on using new syringes and needles during injections or medical procedures?**
   - ☐ Yes
   - ☐ No
2. **Do you avoid sharing personal items such as razors or toothbrushes?**
   - ☐ Yes
   - ☐ No
3. **Do you verify the cleanliness of medical and dental equipment before treatment?**
   - ☐ Always
   - ☐ Sometimes
   - ☐ Never
4. **Do you wear gloves while handling blood or other body fluids at work (if applicable)?**
   - ☐ Yes
   - ☐ No

#### **Section 7: Open-Ended Questions**

1. What challenges do you face in accessing hepatitis screening or treatment services?
   - ☐ Not sure
   - ☐ Lack of healthcare facilities
   - ☐ Financial problems
   - ☐ Lack of screening tests
   - ☐ Lack of awareness
2. What measures do you think the government or healthcare providers should take to control HDV in Pakistan?
   - ☐ No idea
   - ☐ Mass testing program
   - ☐ Awareness campaign
   - ☐ Mass vaccination program
3. In your opinion, what are the biggest barriers to controlling hepatitis in Pakistan?
   - ☐ Not sure
   - ☐ Limited public awareness
   - ☐ Inadequate healthcare facilities
   - ☐ Lack of screening and diagnosis
   - ☐ Poor hygiene
4. How can healthcare providers improve hepatitis awareness in your community?
   - ☐ Don’t know
   - ☐ Through seminar & workshops
   - ☐ social media and television
   - ☐ Arrange awareness campaigns
5. Do you think cultural or societal norms affect how hepatitis is perceived and treated?
   - ☐ Yes
   - ☐ No
