# Supplementary material for: Assessment of self-reported prevalence, vaccination status, knowledge and behavioral determinants of hepatitis B and D in Pakistan: a cross-sectional study
Source: Front Microbiol. 2026 Feb 12;17:1748793. doi: 10.3389/fmicb.2026.1748793 (PMC12935930; doi:10.3389/fmicb.2026.1748793)
Supplement: Supplementary file 2 [file Table_2.docx]

**Table S1:** Risk factor exposures and behavioral practices related to general and family higher self-reported prevalence.

| **S#No** | **Questions** | **Response** | **Family HBV/HDV Prevalence** | | **Chi-Square** | **P-value** |
| --- | --- | --- | --- | --- | --- | --- |
|  |  |  | **–ve (n=771)** | **+ve (n=209)** |  |  |
| 1 | Have you been vaccinated against hepatitis B? | No | 605 | 118 | 41.172 | 0.00 |
|  |  | Yes | 166 | 91 |  |  |
| 2 | Do you have any chronic illnesses (e.g., diabetes, hypertension)? (Please specify) | No | 737 | 184 | 16.574 | 0.00 |
|  |  | Yes | 34 | 25 |  |  |
| 3 | Have you experienced any of the following symptoms in the last 6 months? | None | 475 | 99 | 13.740 | 0.000 |
|  |  | Any one or all | 296 | 110 |  |  |
| 4 | Are you currently undergoing treatment for hepatitis? | No | 767 | 197 | 27.928 | 0.00 |
|  |  | Yes | 4 | 12 |  |  |
| 5 | Have you ever received a blood transfusion? | No | 730 | 186 | 8.712 | 0.04 |
|  |  | Yes | 41 | 23 |  |  |
| 6 | Have you ever undergone any surgical procedures? | No | 557 | 162 | 2.335 | 0.074 |
|  |  | Yes | 214 | 47 |  |  |
| 7 | Have you ever shared needles, syringes, or other medical equipment? | No | 705 | 185 | 1.684 | 0.124 |
|  |  | Yes | 66 | 24 |  |  |
| 8 | Have you ever undergone dental treatment in an unlicensed clinic? | No | 691 | 185 | 0.212 | 0.363 |
|  |  | Yes | 80 | 24 |  |  |
| 9 | Do you frequently visit barbershops or salons for shaving or grooming? | No | 291 | 84 | 0.417 | 0.285 |
|  |  | Yes | 480 | 125 |  |  |
| 10 | Have you received treatment or medical procedures from unlicensed healthcare providers? | No | 638 | 153 | 9.622 | 0.002 |
|  |  | Yes | 133 | 56 |  |  |
| 11 | Do you frequently travel outside your province or country for work or leisure? | No | 528 | 127 | 4.418 | 0.022 |
|  |  | Yes | 243 | 82 |  |  |
| 12 | Have you been diagnosed with hepatitis B, or hepatitis D? | No | 742 | 167 | 65.284 | 0.000 |
|  |  | Yes | 29 | 42 |  |  |
| 13 | Have you ever shared or reused shaving blades at barbershops? | No | 731 | 201 | 0.653 | 0.272 |
|  |  | Yes | 40 | 8 |  |  |
| 14 | Have you lived or worked with someone diagnosed with hepatitis B, or D? | No | 647 | 72 | 205.917 | 0.000 |
|  |  | Yes | 124 | 137 |  |  |
| 15 | Do you wear gloves while handling blood or other body fluids at work (if applicable)? | No | 110 | 24 | 1.080 | 0.178 |
|  |  | Yes | 661 | 185 |  |  |
