# Supplementary material for: Assessment of self-reported prevalence, vaccination status, knowledge and behavioral determinants of hepatitis B and D in Pakistan: a cross-sectional study
Source: Front Microbiol. 2026 Feb 12;17:1748793. doi: 10.3389/fmicb.2026.1748793 (PMC12935930; doi:10.3389/fmicb.2026.1748793)
Supplement: Supplementary file 3 [file Table_3.docx]

**Supply Table S2:** Self-reported prevalence of HBV/HDV across demographic.

| **S. No** | **Variable** | **Unique Variable** | **Status of Hepatitis** | | **Chi-Square** | **P-Value** |
| --- | --- | --- | --- | --- | --- | --- |
|  |  |  | **negative** | **positive** |  |  |
| 1 | Age (Years) | 18-24 | 672 | 23 | 109.04 | 0.000 |
|  |  | 25-34 | 184 | 21 |  |  |
|  |  | 35 and above | 52 | 28 |  |  |
| 2 | Gender | Female | 184 | 20 | 2.285 | 0.090 |
|  |  | Male | 724 | 52 |  |  |
| 3 | Province of Residence | Punjab | 113 | 28 | 62.930^a^ | 0.000 |
|  |  | Khyber Pakhtunkhwa | 95 | 9 |  |  |
|  |  | Baluchistan | 174 | 24 |  |  |
|  |  | Islamabad Capital Territory | 422 | 8 |  |  |
|  |  | Gilgit-Baltistan | 56 | 0 |  |  |
|  |  | Sindh | 48 | 3 |  |  |
| 4 | Residence Type | Urban | 523 | 39 | 0.321 | 0.327 |
|  |  | Rural | 385 | 33 |  |  |
| 5 | Education | Higher | 881 | 44 | 209.733 | 0.000 |
|  |  | Secondary | 24 | 12 |  |  |
|  |  | Primary | 3 | 16 |  |  |
| 6 | Occupation | Healthcare worker | 195 | 28 | 42.614 | 0.000 |
|  |  | Office Worker | 38 | 13 |  |  |
|  |  | Students | 675 | 31 |  |  |
| 7 | Monthly Household Income (PKR) | < 20000 | 81 | 16 | 29.867 | 0.000 |
|  |  | 50001-100000 | 309 | 17 |  |  |
|  |  | > 100000 | 267 | 7 |  |  |
|  |  | 200000-500000 | 251 | 32 |  |  |
| 8 | Family member infected with HBV | No | 742 | 29 | 68.279^a^ | 0.000 |
|  |  | Yes | 166 | 43 |  |  |
